# The impact of infectious diseases consultation on the management and outcomes of *Pseudomonas aeruginosa* bacteraemia in adults: a retrospective cohort study

**DOI:** 10.1186/s12879-021-06372-5

**Published:** 2021-07-09

**Authors:** Fabian Chiong, Mohammed S. Wasef, Kwee Chin Liew, Raquel Cowan, Danny Tsai, Yin Peng Lee, Larry Croft, Owen Harris, Stella May Gwini, Eugene Athan

**Affiliations:** 1 Department of Medicine, Alice Springs Hospital, PO Box 2234, Alice Springs, NT 0871 Australia; 2 Bankstown-Lidcombe Hospital, Sydney, Australia; 3 Department of Infectious Diseases, University Hospital Geelong, Geelong, Australia; 4 Australian Clinical Laboratories, Geelong, Australia; 5 University of Queensland Centre for Clinical Research, University of Queensland, Brisbane, Australia; 6 Rural and Remote Health NT, Flinders University, Alice Springs, NT Australia; 7 Deakin Genomic Centre, Deakin University, Geelong, Australia; 8 School of Life and Environmental Sciences, Deakin University, Geelong, Australia; 9 Asian Institute of Medicine, Science and Technology University, Bedong, Kedah Malaysia; 10 University Hospital Geelong, Geelong, Australia; 11 School of Medicine Deakin University, Geelong, Australia

**Keywords:** *Pseudomonas aeruginosa*, Bacteraemia, Infectious diseases consultation, Mortality

## Abstract

**Background:**

*Pseudomonas aeruginosa* bacteraemia (PAB) is associated with high mortality. The benefits of infectious diseases consultation (IDC) has been demonstrated in *Staphylococcal aureus* bacteraemia and other complex infections. Impact of IDC in PAB is unclear. This study aimed to evaluate the impact of IDC on the management and outcomes in patients with PAB.

**Methods:**

This is a retrospective cohort single-centre study from 1 November 2006 to 29 May 2019, in all adult patients admitted with first episode of PAB. Data collected included demographics, clinical management and outcomes for PAB and whether IDC occurred. In addition, 29 *Pseudomonas aeruginosa* (PA) stored isolates were available for Illumina whole genome sequencing to investigate if pathogen factors contributed to the mortality.

**Results:**

A total of 128 cases of PAB were identified, 71% received IDC. Patients who received IDC were less likely to receive inappropriate duration of antibiotic therapy (4.4%; vs 67.6%; *p* < 0.01), more likely to be de-escalated to oral antibiotic in a timely manner (87.9% vs 40.5%; *p* < 0.01), undergo removal of infected catheter (27.5% vs 13.5%; *p* = 0.049) and undergo surgical intervention (20.9% vs 5.4%, *p* = 0.023) for source control. The overall 30-day all-cause mortality rate was 24.2% and was significantly higher in the no IDC group in both unadjusted (56.8% vs 11.0%, odds ratio [OR] = 10.63, *p* < 0.001) and adjusted analysis (adjusted OR = 7.84; 95% confidence interval, 2.95–20.86). The genotypic analysis did not reveal any PA genetic features associated with increased mortality between IDC versus no IDC groups.

**Conclusion:**

Patients who received IDC for PAB had lower 30-day mortality, better source control and management was more compliant with guidelines. Further prospective studies are necessary to determine if these results can be validated in other settings.

## Background

*Pseudomonas aeruginosa* (PA) is a ubiquitous environmental Gram-negative pathogen that is responsible for many opportunistic and healthcare associated infections. *Pseudomonas aeruginosa* is the third most common Gram-negative pathogen causing bacteraemia in Australia [1]. It is also a common pathogen causing nosocomial infections such as pneumonia, postoperative wound infections and urinary tract infections (UTI) [2, 3]. *Pseudomonas aeruginosa* bacteraemia (PAB) is associated with prolonged hospital stays and has high mortality rates, particularly in neutropenic and immunocompromised hosts [1, 4]. It is clinically indistinguishable from other Gram-negative bacterial infections and given its intrinsic antimicrobial resistance, empiric therapy may be ineffective [5].

Data gathered across 36 institutions in Australia in the 2017 Australian Group on Antimicrobial Resistance (AGAR) study found that PA accounted for 8.8% of a total 7910 reported cases of Gram-negative bacteraemia [1]. Of the patients with PAB, 13.9% had a length of stay of more than 30 days [1]. This is relative to 46.3% of patients with Enterobacterales bacteraemia who were discharged within 7 days [1]. The 30-day mortality for the cohort of patients with PAB was 20.7% [1]. Studies conducted prior to availability of effective therapies in the 1960s showed a mortality rate as high as 90% [6, 7]. With the availability of more effective therapy, recent studies have shown mortality rates ranging between 18 and 61% [1, 4, 8–12].

Infectious diseases specialist consultation (IDC) is associated with improved clinical outcome in patients with *Staphylococcal aureus* bacteraemia, resulting in reduced mortality [13–17], lower treatment failure rates [18], better adherence to guidelines [13, 15], lower antimicrobial resistance development [15], higher probability of identifying a removable focus of bacteraemia [14, 16] and higher likelihood of patients receiving appropriate empiric and directed antimicrobial therapies for an appropriate duration [14, 15]. Infectious diseases specialist consultation was also shown to reduce mortality in multidrug-resistant organism infection [19], candidemia [20, 21], cryptococcal infection [22] and enterococcal bacteraemia [23, 24].

In this retrospective study, we aimed to evaluate the impact of IDC on the clinical management and outcome of patients with PAB, including the 30-day mortality. We also aimed to identify associations between 30-day mortality and patient factors, presence of IDC, source of PAB, PA pathogen phenotypic and genotypic factors.

## Methods

Ethics for this study was approved by the University Hospital Geelong Health Research and Ethics Committee (No.14/86).

### Study design

We performed a retrospective study on consecutive adult patients admitted to the hospital with PAB from 1 November 2006 to 29 May 2019. In addition, 29 PA isolates from 29 patients which had been stored between 11 April 2017 and 29 May 2019 were examined to investigate whether pathogen factors contributed to the mortality, using Illumina Novaseq whole genome sequencing.

### Setting

The University Hospital Geelong is the major regional tertiary-care centre for South Western Victoria, Australia, catering to a population of approximately 600,000 people. It has 370 acute inpatient beds, an integrated Haematology, Oncology and Radiation Oncology service and a 24-bed, level 3, Intensive Care Unit (ICU).

### Patients

All inpatients aged 18 years or over with at least one positive blood culture for PA during the study period were included. Detailed medical records for the corresponding admission were cross-referenced for data on age, gender, date of admission, date of diagnosis of PAB, date of discharge, date of death, underlying comorbidities, any invasive procedures (surgery, gastrointestinal and urological instrumentation) performed 30 days prior to positive blood culture for PA, source of their PAB (if known), appropriateness of their antimicrobial therapy, source control intervention, bedside consultation by infectious diseases (ID) specialist and 30-day mortality.

The pre-morbid and concurrent disease burden for each patient was assigned a value for severity using the Charlson comorbidity index [25]. This score was subsequently used to risk stratify the cohort and measure the impact of comorbid disease on 30-day mortality.

### Microbiological testing

Blood cultures were incubated in BD BACTEC™ Instrumented Blood Culture System and microorganisms from positive blood cultures were processed via standard method on horse blood, chocolate and MacConkey agar plates. Species identification was processed by Bruker MALDI-TOF or VITEK (bioMérieux) machine and antimicrobial susceptibility testing was processed by VITEK (bioMérieux) machine.

DNA extraction of this isolate was performed using the HiYieldTM Genomic DNA Mini Kit and Nextera library preps were made for each sample according to the standard protocol. The libraries were subjected to whole-genome sequencing using the NovaSeq 6000 Sequencer (Illumina) according to the manufacturer’s instructions. Approximately 2Gb of 2x150nt paired-end reads were generated for each sample. Reads were cleaned and trimmed using fastp with default parameters. Snippy (https://github.com/tseemann/snippy v4.6.0) was used to call SNPs based on the reference PA01 genome. SNP frequencies were extracted from the Snippy output with custom perl scripts and Fisher’s exact test was run for each SNP contingency matrix using Matlab R2019b. Gene presence/absence across all samples was determined based on 95% identity over 90% of the length of the protein using Diamond blastp (v2.0.4). Presence/absence was counted per isolate and summed for the no IDC and IDC groups. A Fisher exact test was run in Matlab for each of the 177,085 proteins and variants to see if any were significantly present between the 2 groups.

### Clinical definitions

- *Pseudomonas aeruginosa* bacteraemia episode was defined as a patient with consistent clinical illness having PA isolated from one or more of the aerobic or anaerobic bottles of their blood culture. The date of onset of PAB was defined as the date of the first positive blood culture for PA.
- Fever was defined as a single recorded body temperature ≥ 38 °C.
- A systolic blood pressure of less than 90 mmHg was attributed to a presentation with shock.
- A patient was deemed to have febrile neutropenia if they had a temperature of 38 °C or over and an absolute neutrophil count less than 0.5 × 10^9^/L.
- Health care associated infection was defined by a positive blood culture of PA more than 48 h after hospital admission, or 3 days after discharge, or less than 30 days after an invasive procedure.
- The criterion for being considered immunosuppressed was any form of chemotherapy or immunomodulatory medications (equivalent or more than 20 mg of prednisolone a day) for longer than 4 weeks, patients with a clinical diagnosis of hematologic malignancy, or HIV infection, or has received solid organ or bone marrow transplantation.
- Duration of antibiotic treatment for PAB was dependent on the source of infection and appropriateness was assessed according to the Australian Therapeutic Guidelines: Antibiotic version 16, 2019 [26] and antimicrobial susceptibility testing. Compliance assessment was done by ID specialist, FC.
- De-escalation to oral antibiotic (Ciprofloxacin) in a timely manner was assessed according to the Australian Therapeutic Guidelines: Antibiotic version 16, 2019 [26]. Compliance assessment was done by ID specialist, FC.
- Heart failure was defined as left ventricular ejection fraction of 40% or less with at least class II New York Heart Association functional status.
- Liver cirrhosis was defined by the presence of radiological features of liver cirrhosis on abdominal ultrasound or documented diagnosis of liver cirrhosis in the patient’s clinical notes.
- Renal insufficiency was defined as chronic kidney disease stage 3 or more using glomerular filtration rates and albumin creatinine ratio categories.
- Infectious diseases consultation included bedside consultation by a member of the ID service with entry of comments and recommendations for further clinical management were entered in the inpatient notes. Infectious diseases consultation was readily available and performed upon request from the primary service doctor with expectation for IDC to take place within 24 h of request.

### Outcomes

Primary outcomes: Impact of IDC on all-cause 30-day mortality, clinical management and outcomes in patients with PAB.

Secondary outcome: Associations of 30-day all-cause mortality in patients with PAB, including patient factors, presence of IDC, source of PAB, PA pathogen phenotypic and genotypic factors.

### Statistical analysis

Categorical variables were reported as frequencies and percentages, continuous variables were summarised using median with interquartile range or mean with standard deviation. Patient characteristics considered were age (categorised using cut-offs of 65 and 75 years), Charlson comorbidity index (dichotomised using cut-off = 5), febrile neutropenic status, immunosuppression status, haemodynamic status, presence of IDC, source of infection, appropriateness antibiotics therapy. Categorical variables were analysed with Fisher’s exact test, and continuous variables were analysed with Wilcoxon rank-sum test when appropriate. The relationship between patient characteristics and mortality were assessed using logistic regression and effects were reported as odds ratios (OR) with 95% confidence interval. The adjusted logistic regression model accounted for variables that were of presumed clinically importance (age) and with *p* < 0.20 in the unadjusted model. Survival rates in patients who received ID bedside consultation (+IDC) and those who did not (−IDC) were compared using the Log-rank test and cox proportional hazard model to account for confounding factors. Kaplan-Meier survival curves were constructed to illustrate the differences between the two groups. Logistic and cox regression adjusted for age, Charlson comorbidity index, IDC, surgical intervention and appropriateness of antimicrobial therapy. Fisher’s exact test and correcting for multi-testing for single nucleotide polymorphisms analyses were undertaken using Stata Statistical Software version 15 (StataCorp, 2015. The College Press, Texas).

## Results

### Demographics

A total of 128 patients with PAB were identified, of whom 91 (71%) received IDC and Table 1 summarises the demographic characteristics and present comorbidities. The median age of this cohort was 68 years old with almost an equal number of males and females. Baseline demographics of +IDC and –IDC were largely similar with exception for the proportion of patients with a Charlson comorbidity index of more than 5, which was larger in the –IDC group (54.9% vs 75.5%, *p* = 0.048). The difference among individual comorbidities such as haematological malignancy, solid organ tumour, liver cirrhosis, renal insufficiency, heart failure, immunosuppression between the 2 groups were all less than 10% except for diabetes, which had 10% more in the –IDC group (24.3% vs 14.3%; *p* = 0.269). The admitting team distribution among the 2 groups is similar except for general medicine, where there was 11.5% more in the –IDC group (32.4% vs 20.9%; *p* = 0.248).

**Table 1:** Demographics of patients with *Pseudomonas aeruginosa* bacteraemia

	All patients (*n* = 128)	+IDC (*n* = 91)	-IDC (*n* = 37)	*P* Value
Age (years): Median	68	62	66	0.203
IQR	59–78	43–75	47–77	
**Male:** n (%)	69 (53.9)	51 (56.0)	18 (48.6)	0.572
**Comorbidities and medical history:** n (%)				
Charlson comorbidity index (severe, score ≥ 5)	78 (60.9)	50 (54.9)	28 (75.7)	0.048
Haematological malignancy	46 (35.9)	35 (38.5)	11 (29.7)	0.465
Solid organ tumour	28 (21.9)	18 (19.8)	10 (27.0)	0.507
Diabetes	22 (17.2)	13 (14.3)	9 (24.3)	0.269
Liver cirrhosis	15 (11.7)	12 (13.2)	3 (8.1)	0.612
Renal insufficiency	25 (19.5)	20 (22.0)	5 (13.5)	0.396
Heart failure	9 (7.0)	4 (4.4)	5 (13.5)	0.148
Immunosuppressed	86 (67.2)	59 (64.8)	27 (73.0)	0.496
**Admitting Teams**: n (%)				0.455
Hematology	48 (37.5)	36 (39.6)	12 (32.4)	
General medicine	31 (24.2)	19 (20.9)	12 (32.4)	
Medical oncology	26 (20.3)	18 (19.8)	8 (21.6)	
Renal medicine	5 (3.9)	3 (3.3)	2 (5.4)	
Urology	5 (3.9)	5 (5.5)	0 (0)	
Other ^a^	13 (10.2)	9 (9.9)	4 (10.8)	

*Abbreviations*: *IQR* interquartile range, *+IDC* received infectious diseases specialist consultation, *−IDC* did not receive infectious diseases specialist consultation

^a^ Other group includes General surgery, Gastroenterology, Cardiology, Cardiothoracic, Neurology, Orthopaedics, Radiation Oncology and Vascular teams

### Clinical management and outcomes

Clinical management and outcomes observed for +IDC versus –IDC groups are presented in Table 2. Approximately half (49.2%) of all the patients in this cohort required ICU admission, more in the -IDC group were admitted to ICU (62.2% vs 44.0%, *p* = 0.163). A total of 78 episodes (60.9%) of PAB were hospital acquired and the +IDC group had 13.2% more hospital-acquired PAB than the -IDC group (64.6% vs 51.4%, *p* = 0.248). The mean length of stay in hospital was longer in the +IDC group (16.7 vs 12.9 days, *p* = 0.248) although the difference was not statistically significant. The +IDC group was less likely to receive inappropriate or no antibiotic (26.4% vs 43.2%, *p* = 0.06). The +IDC group was also less likely to have inappropriate duration of antibiotics treatment based on the source of infection and treatment of PAB was more frequently in line with the Australian Therapeutic Guidelines: Antibiotic [26] (4.4% vs 67.6%, *p* < 0.001). The +IDC group also had more than twice the proportion of the patients being de-escalated to oral antibiotic in a timely manner compared to the –IDC group (87.9% vs 40.5%; *p* < 0.001).

**Table 2:** Clinical Management and Outcomes in *Pseudomonas aeruginosa* bacteraemia

	All patients (*n* = 128)	+IDC (*n* = 91)	-IDC (*n* = 37)	*P* Value
30-day mortality: n (%)	31 (24.2)	10 (11.0)	21 (56.8)	< 0.001
Intensive care unit admission: n (%)	63 (49.2)	40 (44.0)	23 (62.2)	0.163
Acquisition of PAB: n (%)				0.223
Community acquired	50 (39.1)	32 (35.2)	18 (48.6)	
Hospital acquired	78 (60.9)	59 (64.8)	19 (51.4)	
Length of stay in hospital (days; mean, standard deviation)	15.5, 7.8	16.7, 8.5	12.9, 5.7	0.248
Inappropriate or no antibiotic^a^: n (%)	40 (31.3)	24 (26.4)	16 (43.2)	0.060
Inappropriate duration of antibiotic: n (%)	29 (22.7)	4 (4.4)	25 (67.6)	< 0.001
De-escalation to oral antibiotic in a timely manner: n (%)	95 (74.2)	80 (87.9%)	15 (40.5%)	< 0.001
Source of PAB: n (%)				0.786
Unidentified	41 (32.0)	28 (30.8)	13 (35.1)	
Identified	87 (68.0)	63 (69.2)	24 (64.9)	
Intravascular catheter	32 (25.0)	26 (28.6)	6 (16.2)	
Pulmonary infection	20 (15.6)	11 (12.1)	9 (24.3)	
Urinary tract infection	18 (14.1)	14 (15.4)	4 (10.8)	
Skin and soft tissue infection	13 (10.2)	10 (11.0)	3 (8.1)	
Other	4 (3.1)	2 (2.2)	2 (5.4)	
Removal of infected catheter: n (%)	30 (23.4)	25 (27.5)	5 (13.5)	0.049
Surgical intervention for source control: n (%)	21 (16.4)	19 (20.9)	2 (5.4)	0.023

*Abbreviations*: *PA Pseudomonas aeruginosa*, *PAB Pseudomonas aeruginosa* bacteraemia, *+IDC* received infectious diseases specialist consultation, *−IDC* did not receive infectious diseases specialist consultation. ^a^Only one person did not receive antibiotic as per patient’s advanced care plan in the event of septic shock

Source of infection was identified in about two-thirds of patients with a slightly higher rate in the +IDC group (69.2% vs 64.9%, *p* = 0.630). Intravascular catheter was the most frequently identified source of PAB in the +IDC group, 12.4% more than the –IDC group (28.6% vs 16.2%, *p* = 0.216). The most commonly identified source in the –IDC group was pulmonary (24.3%). Infected intravascular catheter was five times more likely to be removed from the patient if the patient received IDC. Patients in +IDC group were also more likely to undergo surgical intervention to remove the source of infection or for source control for PAB (Table 2).

### Mortality

**Fig. 1:**
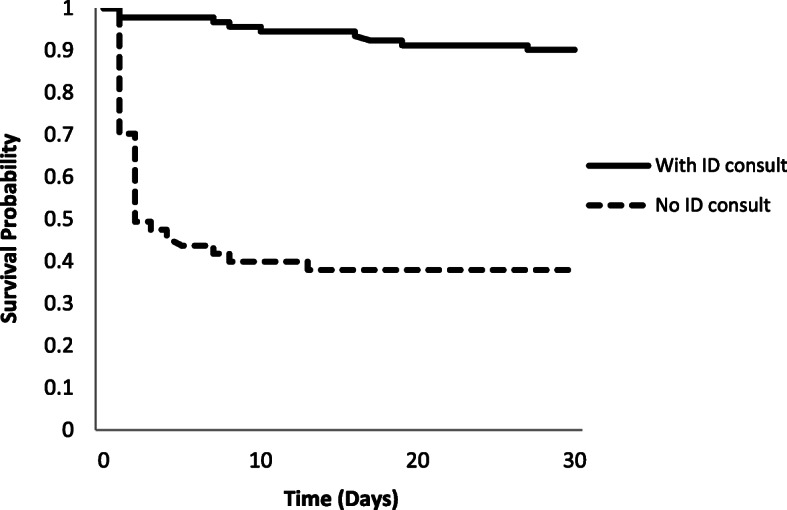
Kaplan-Meier survival curve showing survival probabilities for patients with *Pseudomonas aeruginosa* bacteraemia with +IDC and -IDC

A total of 31 of the 128 patients (24.2%) died within 30-days from the onset of PAB, with a significantly higher mortality rate in the –IDC group (56.8% vs 11.0%) (Table 2). After adjusting for other factors likely to influence 30-day all-cause mortality, the –IDC group was associated with 7-fold increased likelihood of death within 30 days from the time of PAB diagnosis. A pulmonary source was associated with higher rates of 30-day mortality (Table 3), even after controlling for other patient characteristics. *P. aeruginosa* bacteraemia resulting from an intravascular catheter, however, had an inverse association with 30-day mortality (OR 0.15, *p* = 0.015) (Table 3). Other associations with 30-day mortality (based on unadjusted analysis) are presented in Table 3. However, in the adjusted model, the effect of age and Charlson comorbidity index were attenuated (Table 3). Neither neutropenia nor immunosuppression were significantly associated with mortality. Kaplan-Meier survival curves (Fig. 1) clearly showed lower survival probability in patients with -IDC (unadjusted HR 7.45; 95% CI 3.61–15.41; *p* < 0.001 and adjusted HR = 5.58; 95% CI 2.58–12.06; *p* < 0.001).

**Table 3:** Analysis of factors likely to influence 30-day all-cause mortality

Variables	Mortality n/Total (%)	Unadjusted OR (95% CI)	*P* value	Adjusted^a^ OR (95% CI)	*P* value
**IDC**					
Yes	10/91 (11.0)				
No	21/37 (56.8)	10.63(4.20–26.89)	< 0.001	7.84 (2.95–20.86)	< 0.001
**Age** (years)					
< 65	10/53 (18.9)				
≥ 65	21/75 (28.0)	1.67 (0.71–3.94)	0.239	0.69 (0.19–2.44)	0.562
**Age** (years)					
< 75	15/86 (17.4)				
≥ 75	16/42 (38.1)	2.91 (1.26–6.74)	0.012	1.56 (0.50–4.87)	0.441
**Gender**					
Female	13/59 (22.0)				
Male	18/69 (26.1)	1.25 (0.55–2.84)	0.595	1.54 (0.56–4.28)	0.407
**Charlson comorbidity index**					
Score < 5	5/50 (10.0)				
Score ≥ 5	26/78 (33.3)	4.50 (1.59–12.75)	0.005	5.14 (1.22–21.58)	0.025
**Appropriate antibiotic therapy**					
Yes	17/88 (19.3)				
No	14/40 (35.0)	0.44 (0.19–1.03)	0.059	0.38 (0.06–2.26)	0.289
**Surgical intervention for source control**	2/21 (9.5)				
	29/107 (27.1)	3.53 (0.77–16.22)	0.105	2.64 (0.44–15.77)	0.287
**Febrile neutropenia**					
No	22/83 (26.5)				
Yes	9/45 (20.0)	0.69 (0.29–1.67)	0.415	0.97 (0.33–2.89)	0.963
**Immunocompromised**					
No	11/45 (24.4)				
Yes	20/83 (24.1)	0.98 (0.42–2.29)	0.965	0.64 (0.19–2.14)	0.474
**Haemodynamic status at presentation**					
No shock	2/63 (3.2)				
In shock	29/65 (44.6)	24.57 (5.50–109.76)	< 0.001	45.77 (10.30–203.41)	< 0.001
**Source of bacteraemia** ^**b**^					
Pulmonary	11/20 (55.0)	5.38 (1.96–14.76)	0.001	3.32 (0.96–11.46)	0.058
Invasive intravascular catheters	2/32 (6.3)	0.15 (0.03–0.69)	0.015	0.18 (0.04–0.90)	0.036
Urinary tract	1/18 (5.6)	0.16 (0.02–1.24)	0.079	0.17 (0.02–1.32)	0.091
Skin & soft tissue	2/13 (15.4)	0.54 (0.11–2.59)	0.441	0.88 (0.11–6.99)	0.907
Unknown	14/41 (34.2)	2.14 (0.92–4.94)	0.076	1.73 (0.67–4.44)	0.256

*Abbreviations*: *OR* odds ratio, *CI* confidence interval, *ID* infectious diseases specialist

^a^ Adjusted for age, Charlson comorbidity index, IDC, surgical intervention and appropriateness of antimicrobial therapy

^b^ The odds ratio compares odds of death in current group with the odds of death in all other groups combined

### Pathogen phenotypic and genotypic features

**Fig. 2:**
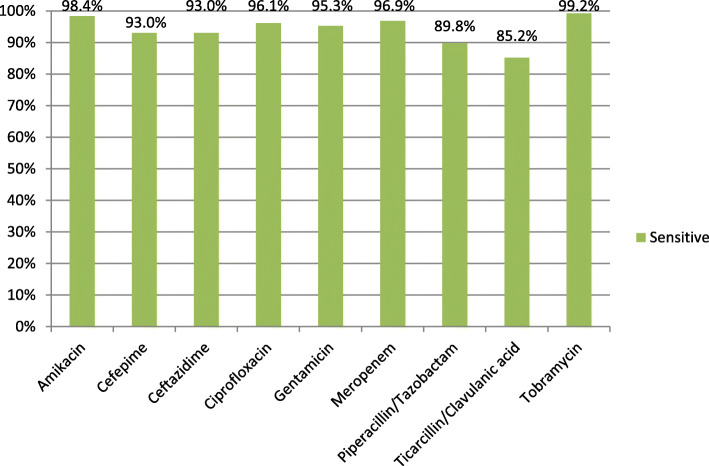
Antibiogram of *Pseudomonas aeruginosa* bacteraemia

All 128 PA isolates showed high proportion of susceptibility to anti-pseudomonal antibiotics. Antibiotic susceptibilities across this cohort of patients were: piperacillin/tazobactam 89.9%, ceftazidime 93.0%, meropenem 96.9%, gentamicin 95.3%, and ciprofloxacin 96.1% (Fig. 2). There were only 2 multidrug-resistant and one extensively drug-resistant PA isolates among all cases.

**Fig. 3:**
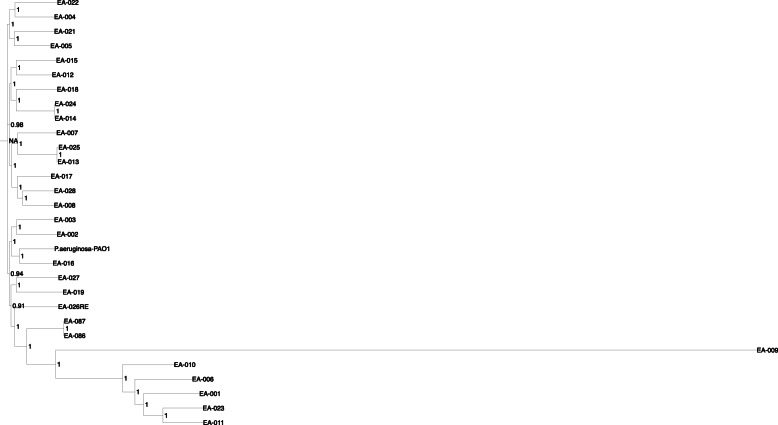
Phylogenetic tree of *Pseudomonas* species

Phylogenomic analysis (Fig. 3) using all public PA genomes and the 29 sequenced isolates showed each PA isolate to be closest to a public genome geographically dispersed with worldwide distribution. Only one isolate was closest to a regionally derived public genome (Melbourne, Australia). This result suggested that there was no common source of patient infection and also the known diversity and lack of population structure found in small organisms, including eukaryotes. Each PA had up to 200 antibiotic resistance genes in its genome. Using a Fisher’s exact test and correcting for multi-testing for each of the 116,941 single nucleotide polymorphisms (SNPs), there was no SNPs with statistically significant allele frequency between the +IDC and –IDC groups or in terms of mortality.

## Discussion

In our retrospective single-centre cohort study, not receiving an IDC was associated with a seven-fold increased risk of 30-day all-cause mortality. This finding is consistent with the results of another study on PAB which was conducted along with methicillin-resistant *Staphylococcal aureus* and candida blood stream infection, where –IDC was associated with 3.2 times increased hazard of death at 3 months and 5.3 times increased hazard of death in hospital compared with persons with IDC [27]. To the best of the authors’ knowledge, the current study is the first that focuses specifically on the impact of IDC on the clinical management and outcomes for PAB.

In addition to the mortality benefit, this study also showed that IDC was associated with improved adherence to guidelines. Patients were less likely to receive inappropriate duration of antibiotic therapy according to the source of PAB and were more likely to be de-escalated to oral therapy in a timely manner. Even though IDC did not increase the likelihood of source identification of PAB, it was associated with better source control. Infected intravascular catheters were more likely to be removed and patients were more likely to have surgical intervention to address the source of PAB in the +IDC group. These factors could be the reasons for the positive impact of ID bedside consultation on the lower 30-day mortality rates in patients with PAB. These findings are also consistent with the benefits of IDC demonstrated in *Staphylococcal aureus* bacteraemia, where clinical management was more adherent to guidelines [13, 15] and higher probability of withdrawing a removable focus of bacteraemia [14, 16].

A previous study failed to demonstrate a mortality benefit of IDC in multidrug-resistant pseudomonal infection; however, patients with multidrug-resistant *Pseudomonas* were one of the smallest groups in the study and the study was underpowered to detect a possible mortality difference [19]. This study also did not specifically examine PAB and only multidrug-resistant pseudomonal infections [19]. Another study found that multiple IDC, not a single consultation, were associated with lower mortality in bacteraemia secondary to both Gram-positive and Gram-negative pathogens [28]. Patients seen by ID specialists are usually more complex, one consultation may not be adequate to resolve the complex medical issues and thus it is crucial for ongoing follow-up with IDC to achieve better patient outcomes [29]. In addition to improved mortality, ID services in the healthcare system were also shown to improve transition of care from inpatient to outpatient based therapies, reduce hospital length of stay, reduce nosocomial infections and thereby lower health care expenditure [30].

We also tested for any pathogen genomic factors which could have affected the results. There were no statistically significant associations in the SNP allele frequencies for the two groups (+IDC versus –IDC and survived versus died), even though there was sufficient statistical power to find these if they were present. The lack of genomic markers showing differences between the groups strongly suggest that there is no genomic or strain which confounds the difference in mortality between the +IDC and -IDC groups.

We noted that the proportion of patients admitted to ICU and with Charleson comorbidity index score ≥ 5 is greater in the –IDC group compared with the +IDC group. This could be explained by the regular multidisciplinary meetings between the ID and ICU teams, which occurred two to three times weekly during the study period. These meetings occurred as bedside discussion as part of the antimicrobial stewardship activities and not considered formal infectious diseases consultation. However, if the patient is deemed too complex for bedside advice, the ICU doctors could request a formal infectious diseases consultation. These multidisciplinary meetings may have reduced the frequency of formal IDC. Patients admitted under haematology, oncology and general medicine units comprise of the top three admitting teams for this cohort. Patients admitted under these teams usually have a higher Charlson’s comorbidity score as most of the diagnoses in the Charlson comorbidity index are cared for under these three teams. There was also a once a week multidisciplinary meeting between the haematology, oncolology and ID teams. Many of the ID specialists also worked as general medicine specialists. Both of these factors could again reduce the frequency of IDC.

### Other associations of all-cause 30-day mortality

Intravascular catheters were the primary source of infection for the highest identified proportion of the cases, at 25%. The unadjusted results also showed a significantly lower mortality rate in this group. This low mortality was likely attributed to the fact that source control was easier to achieve with early removal of infected catheters. Pulmonary sources of PAB had the highest 30-day mortality risk. This is in line with the poor prognostic outcomes observed in patients with PA pneumonia in previous studies [31, 32] The two studies by Arancibia et al. and Charles et al. suggested that the high mortality rates observed in this group was likely due to the greater burden of disease, underlying severe obstructive airways disease and immunosuppression in individuals who were likely to contract opportunistic PA pneumonia.

Variables that we found with a significant association with all-cause 30-day mortality were similar to those previously reported. A 2003 study by Kang et al. identified severe sepsis, pneumonia, delay in starting effective antimicrobial therapy and high Acute Physiology And Chronic Health Evaluation II (APACHE II) score as significant risk factors contributing to increased mortality with PAB [33]. Another study identified ICU admission, coagulopathy, septic shock, and age greater than 65 years as key factors in increased mortality [34]. The presence of neutropenia was not an independent predictor of 30-day mortality relative to other variables in this study. This is consistent with findings of 2 other PAB series in Korea and China [33, 35]. A possible explanation for this could be due to the rapidly evolving targeted immunotherapies for autoimmune conditions and cancers causing immune dysregulation that is not reflected by absolute neutrophil counts.

### Strength and limitations

From our knowledge, this was the only study that has evaluated the PA pathogen genomic and phenotypic factors in assessing the clinical impact of IDC in PAB, acknowledging the lack of a priori hypothesis in terms of intrinsic pathogenicity factors in PA that hopefully could be addressed through future study. Genotypic analysis is costly and therefore we have only performed genomic studies on 29 frozen PAB isolates. Furthermore, frozen samples are only stored for 2 years in the hospital laboratory, hence isolates prior to April 2017 were not available for genotypic analysis.

This was a single-centre study in an Australian regional tertiary hospital with a limited sample size. The results may therefore not be generalised to other settings, particularly metropolitan tertiary centres with different patient case mix. Collaborating with other centres to achieve a larger cohort may strengthen our generalisability. However, the Barwon region’s population is reasonably large and diverse to resemble the broader Caucasian Australian community [36] and University Hospital Geelong is the only tertiary centre in the region.

Patients with fulminant PA sepsis with high Sequential Organ Failure Assessment or APACHE II scores were at high risk mortality [33, 37, 38], and they could have died prior to IDC. Due to the nature of a retrospective study, data on timing of IDC and early mortality were limited and they were not analysed. Madaline et al. described that early infectious disease consultation, within 12 h of ED triage, was associated with lower mortality in patients with severe sepsis or septic shock [39]. Given the clinical significance of this we suggest to further review the value of ID interventions on PAB in a prospective study.

It is possible that due to the retrospective study design, we were unable to account for unknown confounding variables. For example, the no IDC group in our cohort had higher proportion of patients with multiple co-morbidities and this may have led to discrepancies in goals of care, which could possibly affect overall mortality irrespective of IDC. However, the adjusted models have included all possible confounders available in the retrospective dataset.

## Conclusion

This study demonstrated the positive impact of IDC in the 30-day mortality, clinical management and outcomes of adult patients with PAB. Given the association between higher mortality rates and the absence of bedside IDC demonstrated in our study, we recommend that IDC be considered as standard of care for all adult PAB. Prospective studies on the impact of IDC in the management and outcomes PAB, especially focusing on the timing and barriers to IDC, are warranted to determine if these results can be validated in larger cohort.

## Data Availability

The datasets used and/or analysed in the study are available from the corresponding author on reasonable request.

## Abbreviations

PA: *Pseudomonas aeruginosa*
PAB: *Pseudomonas aeruginosa* bacteraemia
ICU: Intensive Care Unit
ID: Infectious diseases
IDC: Infectious diseases consultation
+IDC: Received infectious diseases specialist consultation
-IDC: Did not receive infectious diseases specialist consultation
